# Dance and Dietary Intervention Improves Metabolic Health, Fitness, and Quality of Life With Modest Gut Microbiota Shifts in Breast Cancer Patients With Obesity: A Pilot RCT

**DOI:** 10.1002/cnr2.70611

**Published:** 2026-06-19

**Authors:** Libuša Nechalová, Adela Penesová, Ivan Hric, Peter Olej, Miroslava Šimiaková, Simona Ugrayová, Jenni Hekkala, Simona Kelčíková, Marián Grendár, Satu Pekkala, Viktor Bielik

**Affiliations:** ^1^ Department of Biological and Medical Sciences Faculty of Physical Education and Sport, Comenius University in Bratislava Bratislava Slovakia; ^2^ Biomedical Center, Institute of Clinical and Translational Research, Slovak Academy of Sciences Bratislava Slovakia; ^3^ Faculty of Sport and Health Sciences, University of Jyväskylä Jyväskylä Finland; ^4^ Department of Public Health Comenius University Bratislava, Jessenius Faculty of Medicine Martin Slovakia; ^5^ Biomedical Center Martin, Jessenius Faculty of Medicine in Martin, Comenius University in Bratislava Martin Slovakia

**Keywords:** beta diversity, body‐image, breast cancer, cardiorespiratory fitness, obesity, physical activity

## Abstract

**Background:**

Post‐treatment weight gain and gut dysbiosis are important concerns in breast cancer patients. However, evidence on the gut microbiota in this population, particularly in relation to physical activity, is limited.

**Aim:**

Therefore, we compared gut microbiota in breast cancer patients with obesity with healthy controls and non‐cancer controls with obesity and subsequently examined the effects of a combined dance and dietary intervention on gut microbiota, metabolic health, physical fitness, and quality of life.

**Methods and Results:**

The observational part compared gut microbiota in breast cancer patients with obesity (BCO, BMI 32.43 ± 4.90 kg/m^2^) with non‐cancer controls with obesity (OC, BMI 37.78 ± 6.68 kg/m^2^) and healthy controls (HC, BMI 21.26 ± 1.26 kg/m^2^). A controlled trial was conducted in breast cancer patients with obesity, with an intervention group (INT, *n* = 13) receiving a 12‐week combined dance and dietary intervention and non‐intervention controls (CTRL, *n* = 10). Gut microbiota was assessed using 16S rRNA sequencing, physical fitness was evaluated by an incremental bicycle ergometer test and motor tests, and quality of life was measured using the EORTC QLQ‐C30 and BR23 questionnaires. In the observational study, breast cancer patients showed significant differences in beta diversity and a lower relative abundance of health‐associated bacteria (e.g., *Faecalibacterium prausnitzii* ) compared with both controls. In the controlled trial, the intervention led to a significant improvement in body composition, physical fitness (e.g., Vo2max, handgrip strength), and several validated quality‐of‐life domains (e.g., fatigue, body image). A statistically significant difference in beta diversity at the post‐intervention phylum level was observed (*p* = 0.046, *R* ^2^ = 0.11). Microbiota composition within INT shifted toward, increased health‐associated taxa (*Bifidobacterium* spp.) and reduced opportunistic pathogens ( *Klebsiella oxytoca* ). However, a decrease in butyrate‐producing taxa (*Ruminococcus bromii, Ruminiclostridium hungatei*) was also observed.

**Conclusion:**

Breast cancer patients showed more negative shifts in gut microbiota compared with both controls. In addition, a 12‐week combined dance and dietary intervention improved body composition, physical fitness, quality of life, and was associated with mixed but potentially beneficial changes in select gut microbiota taxa among breast cancer patients with obesity.

**Trial Registration:**

Clinical trial registration number: NCT07213271

**Abbreviations**
ALT: alanine aminotransferase
AST: aspartate aminotransferase
BCO: breast cancer patients with obesity
BMI: body mass index
CTRL: non‐intervention control group
EMMeans: estimated marginal means
FTVS UK: Faculty of Physical Education and Sport, Comenius University in Bratislava
GGT: gamma‐glutamyl transferase
HC: healthy controls
HDL: high‐density lipoprotein
HOMA‐IR: homeostatic model assessment of insulin resistance
INT: intervention group
LDL: low‐density lipoprotein
OC: non‐cancer controls with obesity
PCoA: principal coordinate analysis
PERMANOVA: permutational multivariate analysis of variance
Pts: points
r(rb): rank‐biserial correlation
RFmax: maximal respiratory frequency
RMR: resting metabolic rate
SCFA: short‐chain fatty acids
SE: standard error
T‐chol: total cholesterol
TG: triglycerides
VEmax: maximal minute ventilation
VO_2_max: maximal oxygen uptake
WHR: waist‐to‐hip ratio
*ε* ^2^
: epsilon‐squared
*η* ^2^
: eta‐squared

## 1 Introduction

Breast cancer is projected to increasingly contribute to global mortality, with its relative ranking anticipated to rise from 22nd to 16th among all causes of death over the next 30 years, increasing from 0.82 million global deaths in 2022 to 1.16 million by 2050 [1]. Nevertheless, the survival rates among patients with breast cancer continue to increase [2]. Despite substantial advances in the management and treatment of breast cancer, including early diagnosis and screening [3], several adverse effects have been reported, both in subjective appraisals [4] and in objective health conditions, many of which may persist more than 10 years after diagnosis [5]. Weight gain is a commonly observed consequence of treatment and subsequent recovery, resulting from treatment‐related side effects, changes in dietary patterns, and reduced habitual physical activity [6]. Importantly, obesity has been consistently associated with an increased risk of recurrence, reduced overall survival, and worse cancer‐specific outcomes in breast cancer patients [7].

In addition to well‐documented effects on physical condition and fitness, quality of life, and psychological, cognitive, and emotional health, an increasing number of studies in recent years have reported disturbances in the gut microbial composition [8]. Gut microbiota has been associated with the etiology of breast cancer [9], its treatment [10] and post‐treatment recovery [11]. However, most studies characterizing the gut microbiome in breast cancer have relied on comparisons with normal‐weight control groups [12]. Moreover, the literature on gut microbiome recovery in breast cancer patients remains scarce, and various nutritional strategies and modifiers still need to be explored to demonstrate their potential [13].

The findings on physical exercise provide robust evidence for improving mental and physical health [14], immunity [15] and long‐term patient outcomes [16] supporting the survival benefits in post‐diagnosis breast cancer patients. Nonetheless, only a limited body of literature has reported on the relationship between gut microbiome and physical exercise, both in animal models [17] and patients with cancer [18, 19, 20].

To contribute to a better understanding of the role of physical exercise in improving physical and mental health after cancer therapy, we aimed to explore the effects of a 12‐week combined dance and dietary intervention on the gut bacterial community, physical fitness, and quality of life in breast cancer survivors. We hypothesized that breast cancer patients with obesity will exhibit more negative shifts in gut microbiota composition (e.g., decreased gut diversity) compared with both control groups. Moreover, we hypothesized that the combined dance and dietary intervention will lead to improvements in body composition, metabolic health, physical fitness, and quality of life. We further hypothesized that these changes will be accompanied by a favorable shift in gut microbiota composition (e.g., increased Chao1 index) and improved intestinal permeability.

## 2 Methods

### 2.1 Study Design

**FIGURE 1:**
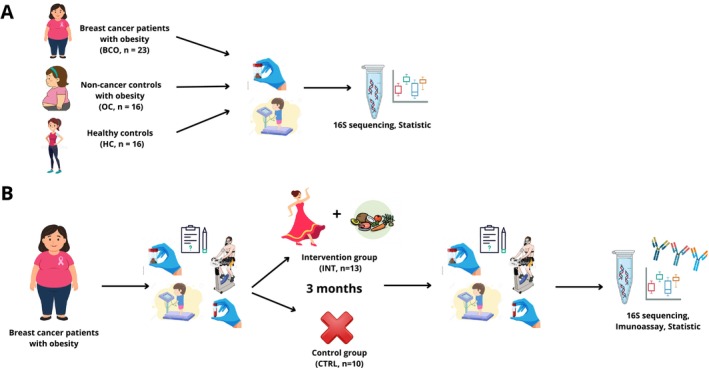
Desing of the study. (A) A cross‐sectional observational study, (B) a randomized controlled trial.

This study consisted of two complementary parts: (a) a cross‐sectional observational study, and (b) a randomized controlled trial. The overall study design is illustrated in Figure 1.

The observational study compared breast cancer patients with obesity in remission (BCO), non‐cancer controls with obesity (OC), and healthy controls (HC). Data collection included stool sample analysis and body composition assessment.

The interventional study consisted of a parallel‐group randomized controlled trial with breast cancer patients with obesity. Data collection comprised blood and stool sample analysis, body composition measurement, physical fitness testing, and completion of questionnaires assessing quality of life, dietary intake, and physical activity before and after intervention. Participants were randomly assigned to study groups using a computer‐generated randomization sequence generated with an online randomization tool (https://www.randomizer.org). Participants were allocated to either (a) an intervention group (INT) participating in a physical exercise and nutrition program or (b) a non‐intervention control group (CTRL). Control participants were instructed to maintain their habitual lifestyle and refrain from initiating any new structured or voluntary physical activity during the study period.

### 2.2 Study Participants and Recruitments

Between April 2025 and June 2025, 40 breast cancer patients with obesity in remission were recruited in a pilot study, which served as the basis for registration as a clinical trial (ClinicalTrials.gov: NCT07213271). The study was conducted at the Faculty of Physical Education and Sport, Comenius University in Bratislava (FTVS UK). Inclusion criteria included female breast cancer patients with obesity who had undergone curative‐intent treatment, including a history of total or partial mastectomy. At the time of enrollment, some patients were receiving adjuvant hormonal therapy, whereas others were not undergoing any active oncological treatment. Remission duration ranged from 1 to 5 years or longer. Of these, patients who completed all required assessments were included in the observational study with non‐cancer controls with obesity and healthy controls. Obesity was defined as a body mass index (BMI) ≥ 30 kg/m^2^ and served as an inclusion criterion for both groups with obesity, while healthy controls were required to have a BMI < 25 kg/m^2^.

Across all groups, exclusion criteria were applied because of their potential influence on metabolic, functional, and psychological outcomes. These included metabolic or endocrine disorders excluding obesity (e.g., uncontrolled diabetes or Cushing's syndrome); severe cardiovascular, musculoskeletal, neurological, or psychiatric conditions limiting safe participation (e.g., unstable angina, advanced arthritis, Parkinson's disease or major depression with functional impairment); acute or chronic inflammatory, autoimmune, or infectious diseases within the past 2 months, or antimicrobial/corticosteroid therapy within the past 3 months.

The trial received ethical approval from the Bratislava Self‐Governing Region Ethics Committee (approval no. 6012/2025/HF‐2). All procedures were conducted in accordance with the Declaration of Helsinki, which provides ethical standards for research involving human participants. Prior to enrollment, each participant provided written informed consent, having first received detailed information about the study protocol and discussed its procedures with the research team.

### 2.3 Combined Dance and Dietary Intervention

Participants in the intervention group attended supervised dance sessions three times per week, each lasting 60 min. Classes were instructed by certified professional dancers and incorporated structured choreographies from three dance genres: folkloric, Latin American, and contemporary. The dance‐based exercise program was implemented at the FTVS UK. The structure and intensity of the dance program have been described in detail in our previous trial [21].

Participants in the intervention group received an individualized nutrition plan with food recipes, generated by nutrition software (Planeat LLC, Bratislava, Slovakia). Total daily energy intake was prescribed according to measured resting metabolic rate (RMR) and ranged from 1200 to 1500 kcal/day. The diet provided 30%–50% of energy from carbohydrates, 25%–30% from fats, 20%–30% from proteins, and 14 g of fiber per 1000 kcal [22]. Energy intake was recorded before the intervention and again during the program (at two‐thirds of its duration).

### 2.4 Data Collection and Measurements

#### 2.4.1 Physical Characteristics

The examinations were carried out at the FTVS UK. Personal and family medical histories were recorded. Body composition measurements included body weight, body fat percentage, muscle mass, visceral fat, and RMR using the InBody 230 device (Serial, USB, LookingBody Basic 120). BMI was calculated as weight in kilograms divided by height in meters squared. Waist and hip circumferences were measured with a flexible anthropometric tape.

#### 2.4.2 Biochemical Assay

Blood samples were collected after overnight fats into tubes with EDTA (for plasma) and without anticoagulant (for serum). After immediate cooling and centrifugation at 4°C, plasma and serum aliquots were stored at −80°C until analysis. Biochemical parameters were measured in a certified clinical laboratory (SYNLAB, Bratislava, Slovakia), as described in [23]. Briefly, serum glucose, alanine aminotransferase (ALT), aspartate aminotransferase (AST), and gamma‐glutamyl transferase (GMT) were analyzed using a Beckman Coulter AU autoanalyzer. Serum insulin was determined by chemiluminescent microparticle immunoassay. Insulin resistance (HOMA‐IR) was calculated as: HOMA‐IR = (fasting plasma glucose × fasting plasma insulin)/22.5. Serum fasting total cholesterol (T‐chol), lipoproteins (LDL, HDL), and triglyceride (TG) levels were determined by enzymatic colorimetric assays on the same autoanalyzer.

#### 2.4.3 Physical Fitness Assessment

Cardiorespiratory fitness was assessed using an incremental test on a bicycle ergometer (Quark CPET, COSMED, Italy). Testing was conducted under standardized environmental conditions (temperature 20°C, relative humidity 50%–60%). Following an initial familiarization period, the warm‐up consisted of 5 min of cycling at a power output of 0.5 w.kg^−1^, with the load increasing by 0.20 w.kg^−1^ every minute until volitional exhaustion. The last step should have been held for an entire minute to record the maximum workload. The aim was to achieve maximum effort in 8–12 min [24]. Maximum exhaustion was confirmed when at least one of the two objective criteria was met: HRmax > 220 minus age or RER peak > 1.10 [25]. During the testing, the procedures were overseen by one medical doctor and one exercise physiologist. The last 10 s of each workload's VO2 data were averaged.

Furthermore, motor performance was evaluated using standardized protocols from the senior fitness test manual [26], including the following: handgrip strength test—maximal isometric grip force was assessed as the best value from three trials per hand using a dynamometer (CAMRY, model EH101, ISO 9001 certified by SGS) in a standing upright position, with the arm moved from the top of the head to the hips during a maximal grip squeeze; 30‐Second Arm Curl Test—upper‐limb muscular endurance was determined by the number of full elbow flexions and extensions completed in 30 s while seated upright, using a 3‐kg dumbbell; and 30‐Second Chair Stand Test—lower‐limb strength and endurance were assessed by the number of complete sit‐to‐stand cycles performed in 30 s from a standardized seated position with arms crossed on the chest.

#### 2.4.4 Dietary Data Collection and Analysis

Dietary intake was assessed both before the intervention and at approximately two‐thirds of the intervention duration using 7‐day self‐reported food diaries. The analysis was conducted using the Planeat LLC database and its online food diary analysis tool (Bratislava, Slovakia; https://planeat.sk). Average daily energy and nutrient intake was calculated based on 7 days of dietary records. Blood and fecal sample collection and food diary completion were carried out within the same week.

Physical activity was evaluated using a validated questionnaire according to Lagerros et al. [27], which records the time spent in different categories of activity (e.g., sedentary behavior, household tasks, walking, cycling, physically demanding work, and sleep/rest). The questionnaire reflected habitual daily physical activity excluding participation in the dance intervention. For the purposes of analysis, activities were further classified into three categories: sedentary, light physical activity, and vigorous physical activity.

#### 2.4.5 Microbial Analysis and Illumina Data Processing

Morning fecal samples were obtained from participants before and after the intervention and frozen and stored immediately at −80°C. Participants received guidance on techniques to avert sample contamination during collection. DNA extraction, library preparation, sequencing, and bioinformatic analysis were performed according to a previously published protocol [28]. Briefly, total DNA was extracted using the ZymoBIOMICS DNA/RNA Kit (Zymo Research, USA). Sequencing libraries targeting the V3–V4 region of the 16S rRNA gene were prepared using the ViennaLab 16S Microbiome NGS Assay (ViennaLab Diagnostics GmbH, Austria). Amplicons were indexed by dual‐index PCR, purified using AMPure XP magnetic beads (Beckman Coulter), fluorometrically quantified, and fragment size distribution was verified using an Agilent TapeStation system (Agilent Technologies, Santa Clara, CA, USA). Libraries were then diluted to 2 nM, pooled equimolarly, and sequenced on the Illumina NextSeq 2000 platform using paired‐end 300 bp reads.

Sequencing data were processed using the ViennaLab NGS Microbiome Assay software package. Raw reads were subjected to quality control and preprocessing using BBMerge [29], Cutadapt [30], and SeqKit [31]. Taxonomic assignment at the species level was performed using the CLARK classification algorithm, which is based on discriminative k‐mer mapping [32]. Reference databases for classification were constructed using sequences from the SILVA (v138.1) and UNITE (v10) databases, while taxonomic annotation was based on the NCBI taxonomy database [33, 34, 35].

#### 2.4.6 Gut Permeability

Serum zonulin (haptoglobin‐2 precursor) levels were measured using a commercial ELISA kit (Human Zonulin ELISA Kit, Thermo Fisher Scientific) according to the manufacturer's instructions. Serum samples were diluted 1:30 and analyzed in duplicate. After incubation on antibody‐precoated plates, detection was performed using a biotinylated antibody and HRP‐conjugated streptavidin, followed by TMB substrate development. The reaction was stopped and absorbance was measured at 450 nm using a SmartReader 96 microplate reader (Accuris Instruments, Edison, NJ, USA). Concentrations were calculated from a four‐parameter logistic (4PL) standard curve and corrected for the dilution factor.

#### 2.4.7 Quality of Life

Quality of life was assessed using the EORTC QLQ‐C30 (version 3.0) and the EORTC QLQ‐BR23 breast cancer module. These internationally validated and widely used instruments assess health‐related quality of life in breast cancer patients and have demonstrated good reliability, validity, and comparability across clinical trials [36]. The EORTC QLQ‐C30 is a validated, standardized questionnaire for measuring health‐related quality of life (HRQoL) in oncology patients. The questionnaire consists of 30 items organized into four domains assessing global health status, five functional subscales (physical, role, emotional, cognitive, and social functioning), core cancer‐related symptoms (fatigue, nausea and vomiting, and pain), and additional single‐item symptoms (dyspnea, insomnia, appetite loss, constipation, diarrhea, and financial difficulties). The EORTC QLQ‐BR23 is a breast cancer–specific module consisting of 23 items that assess symptom burden, body image, sexual functioning, and future perspectives.

#### 2.4.8 Statistical Analysis

All statistical analyses were conducted in R software [37] (v. 4.5.0). Alpha diversity of the gut microbiota was estimated with the Shannon and Chao1 indices. Beta diversity was calculated using the Bray–Curtis dissimilarity index and visualized by principal coordinate analysis (PCoA). Differences in microbiota composition between groups and time points were tested with permutational multivariate analysis of variance (PERMANOVA) based on Bray–Curtis distances with 999 permutations, including age as a covariate. Data normality was assessed using the Shapiro–Wilk test. In the observational study, depending on data distribution, comparisons among the three independent groups were performed using analysis of covariance (ANCOVA) with age as a covariate or the Kruskal–Wallis test, with effect sizes quantified using eta‐squared (*η* ^2^) and epsilon‐squared (*ε* ^2^), respectively. Effect sizes were interpreted as small (*η* ^2^ or *ε* ^2^ ≈ 0.01), medium (≈0.06), and large (≥ 0.14). When a significant main effect was detected, post hoc pairwise comparisons were conducted using Tukey's test following ANCOVA and Dunn's test following the Kruskal–Wallis test, with appropriate adjustment for multiple testing. Significant group differences in the observational study were visualized using violin plots. In the randomized controlled trial, depending on data distribution, within‐group comparisons were analyzed using paired *t*‐tests or the Wilcoxon signed‐rank test, while between‐group comparisons were performed using independent samples *t*‐tests or the Mann–Whitney U test. Effect sizes were calculated using Cohen's d for parametric comparisons and the rank‐biserial correlation (r(rb)) for non‐parametric pairwise comparisons. Effect sizes were interpreted as small (≈0.2), medium (≈0.5), and large (≥ 0.8) for Cohen's d, and small (≈0.1), medium (≈0.3), and large (≥ 0.5) for r(rb). Associations between changes in intestinal permeability (Δ permeability) and changes in selected gut microbial taxa (Δ abundance) were assessed using Spearman's rank correlation coefficients. Reliability of the EORTC QLQ‐C30 and EORTC QLQ‐BR23 scales was evaluated using Cronbach's alpha coefficients. Quality‐of‐life outcomes were analyzed using estimated marginal means (EMMeans) ± standard error (SE), and effect sizes for questionnaire domains were expressed as mean changes in score (pts). Raw EMMeans were linearly transformed to a 0–100 scale according to the EORTC scoring manual. Higher scores indicated better functioning or greater symptom burden depending on the domain, with reversed scaling applied where appropriate. Statistical significance was set at *p* < 0.05. *p*‐values are reported as unadjusted *p*‐values and should be interpreted as exploratory. Sample size was calculated in G*Power based on microbiome alpha diversity, assessed using the Chao1 index as the primary outcome. The expected between‐group difference in change (Δ = 180) was conservatively estimated from that study, which reported an observed difference of approximately 215 units, and the standard deviation (200) was derived from the same study [38]. Using a two‐sided *α* = 0.05 and 80% power, 19 participants per group were required.

## 3 Results

### 3.1 Cross‐Sectional Observational Study

The observational analyses included 23 breast cancer patients with obesity (BCO, mean age 54.39 ± 9.59 years), 16 non‐cancer controls with obesity (OC, mean age 45.00 ± 10.88 years), and 16 healthy controls (HC, mean age 31.38 ± 6.47 years).

#### 3.1.1 Body Composition

Significant differences in body composition were observed among the three observational groups for all assessed parameters (all *p* < 0.001), with large effect sizes for adiposity‐related measures (*ε* ^2^ = 0.42–0.66). Mean BMI was 32.43 ± 4.90 kg/m^2^ in BCO, 37.78 ± 6.68 kg/m^2^ in OC, and 21.26 ± 1.26 kg/m^2^ in HC. Fat percentage reached 43.87% ± 5.79% in BCO, 45.59% ± 6.72% in OC, and 19.81% ± 5.87% in HC, while mean waist circumference was 100.00 ± 12.36 cm, 107.62 ± 11.89 cm, and 69.31 ± 3.28 cm, respectively, and hip circumference 117.07 ± 9.64 cm, 128.38 ± 18.40 cm, and 92.66 ± 4.05 cm, respectively. Significant differences were observed between HC and both BCO and OC in all aforementioned adiposity‐related parameters (all *p* < 0.001). No significant differences were detected between BCO and OC groups, except for BMI (*p* = 0.049).

#### 3.1.2 Gut Microbiota Analysis

Across all samples, the number of reads was 208.5 ± 64.5. Alpha diversity differed significantly among the three observational groups for both the Shannon (*p* = 0.015, *ε* ^2^ = 0.124) and Chao1 (*p* < 0.001, *ε* ^2^ = 0.225) indices (Figure 3A). Significantly higher Shannon diversity and Chao1 richness were observed in HC compared with both BCO and OC (all *p* < 0.01), while no significant differences were detected between BCO and OC for either index (Shannon: *p* = 0.421; Chao1: *p* = 0.342).

**FIGURE 2:**
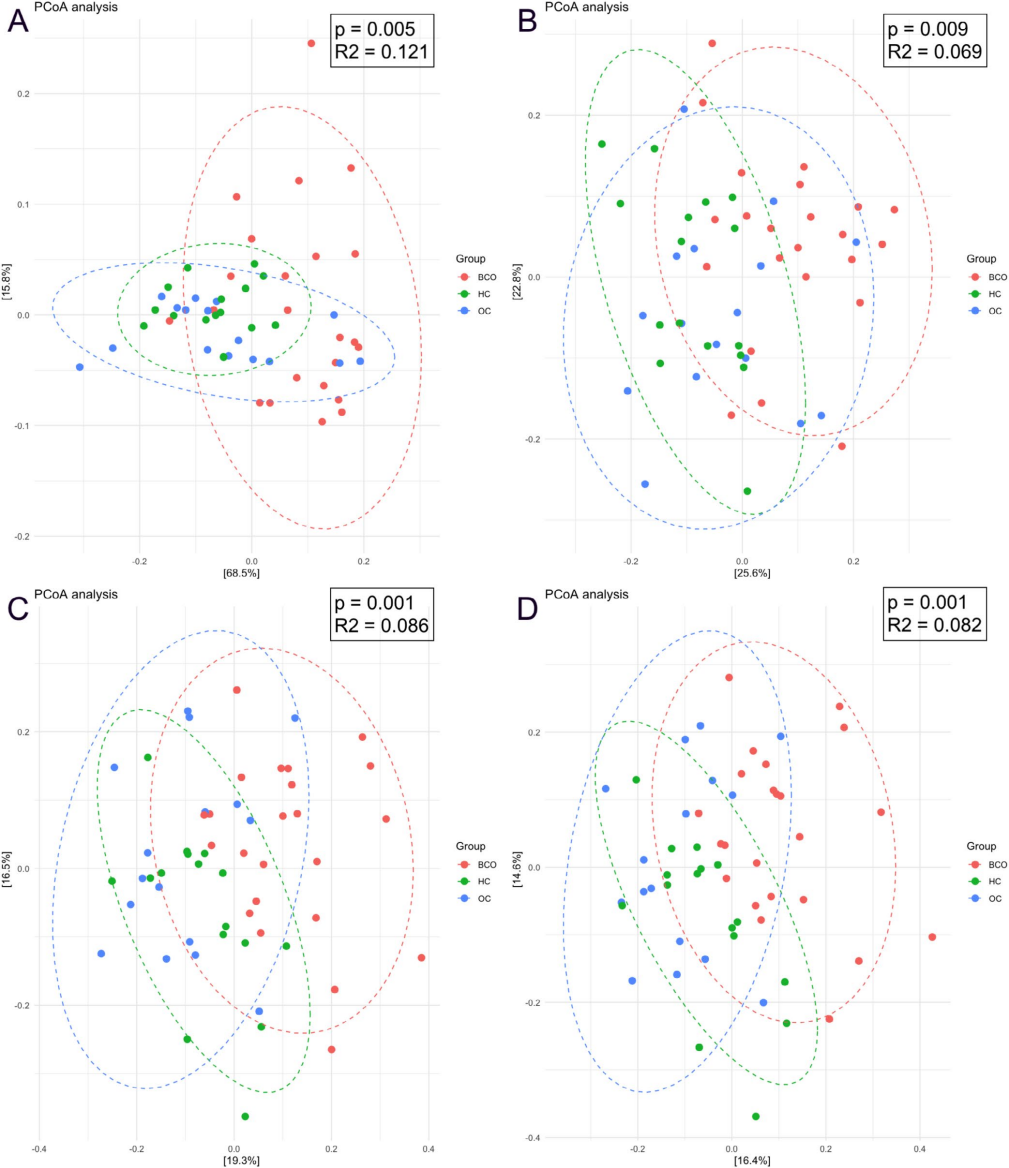
Principal coordinate analysis (PCoA) of gut microbiota beta diversity in the observational study. PCoA plots based on Bray–Curtis dissimilarity illustrate community composition at the phylum (A), family (B), genus (C), and species (D) levels. Each point represents an individual participant, and dashed ellipses indicate 95% confidence intervals for each group. The percentage of variance explained by the first two principal coordinates is shown on the axes. Abbreviations: BCO breast cancer patients with obesity, OC non‐cancer controls with obesity, HC healthy controls.

Beta diversity differed significantly among the three observational groups across phylum, family, genus, and species taxonomic levels (PERMANOVA, all *p* < 0.009; Figure 2). The proportion of variance explained by group membership ranged from 8.2% at the species level to 12.1% at the phylum level. Significant differences were detected by pairwise PERMANOVA between BCO and HC across all taxonomic levels (all adjusted *p* ≤ 0.042, except family level, *p* = 0.12), as well as between BCO and OC across all taxonomic levels (all adjusted *p* ≤ 0.011). In contrast, differences between OC and HC were not significant across all taxonomic levels.

**FIGURE 3:**
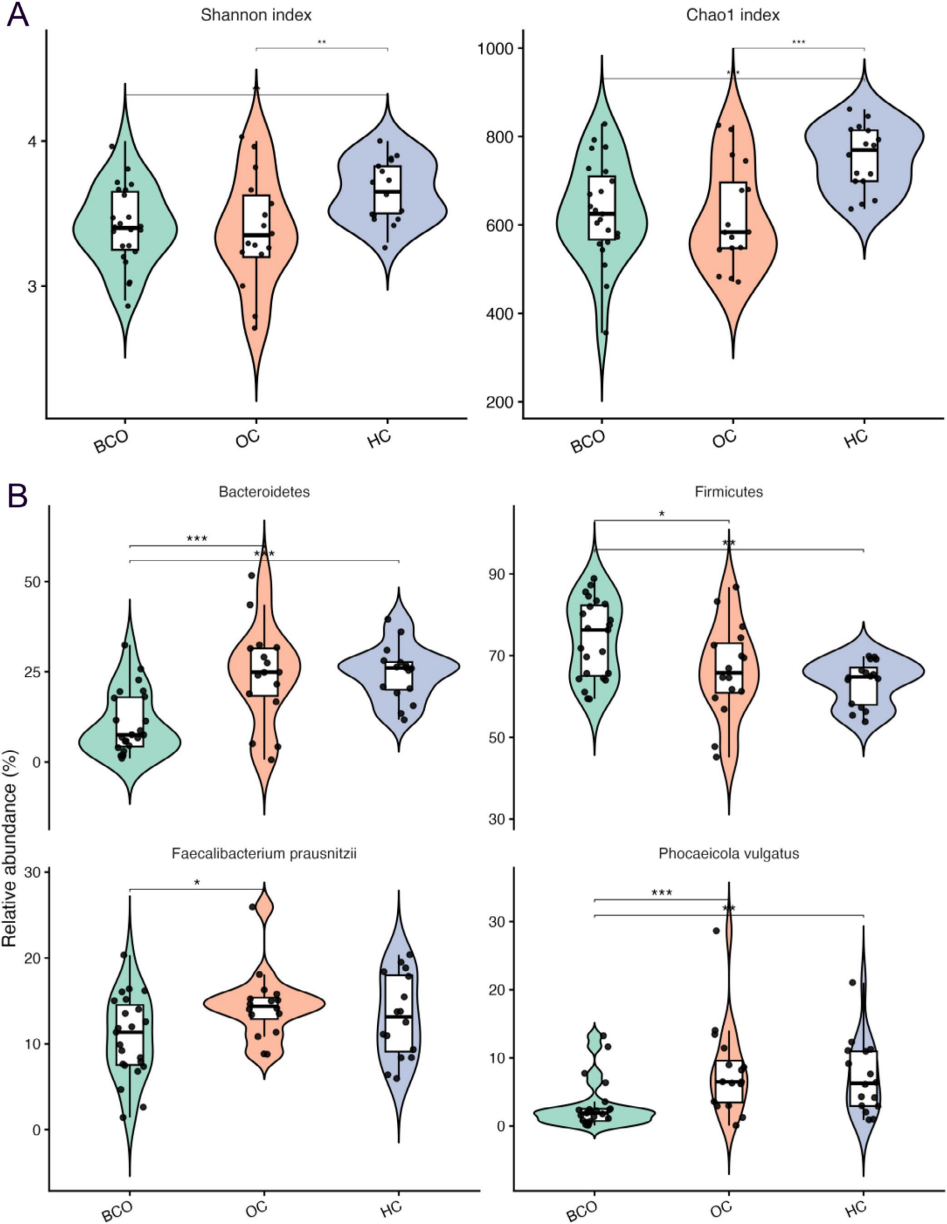
(A) Alpha diversity of the gut microbiota in the observational study groups. Boxplots within violins represent medians and interquartile ranges, with individual data points overlaid. (B) Relative abundance of selected gut microbiota taxa in the observational study. Boxplots within violins represent medians and interquartile ranges, with individual data points overlaid. Abbreviations: BCO breast cancer patients with obesity, OC non‐cancer controls with obesity, HC healthy controls, **p < 0.01, ***p < 0.001.

Significant differences among the three groups were detected across 8 phyla, 33 orders, 65 families, 133 genera, and 224 species. The complete results are provided in Table S1. Among these shifts (Figure 3B), at the phylum level, significant differences were observed for *Bacteroidetes* (*p* < 0.001, *ε* ^2^ = 0.304) and *Firmicutes* (*p* = 0.007, *ε* ^2^ = 0.151). The relative abundance of *Bacteroidetes* was significantly lower in BCO compared with both OC and HC (all adjusted *p* ≤ 0.001), while no difference was detected between OC and HC (*p* = 0.329). Conversely, *Firmicutes* abundance was significantly higher in BCO compared with both OC (*p* = 0.029) and HC (*p* = 0.001), with no significant difference between OC and HC (*p* = 0.142). At the species level, significant differences were identified for *Faecalibacterium prausnitzii* (*p* = 0.043, *ε* ^2^ = 0.058) and *Phocaeicola vulgatus* (*p* = 0.001, *ε* ^2^ = 0.220). The relative abundance of *Faecalibacterium prausnitzii* was significantly lower in BCO compared with OC (*p* = 0.015), with a trend toward lower abundance compared with HC (*p* = 0.084), while no difference was observed between OC and HC (*p* = 0.233). Similarly, *Phocaeicola vulgatus* was significantly less abundant in BCO compared with both OC (*p* = 0.001) and HC (*p* = 0.002), with no significant difference between OC and HC (*p* = 0.370).

### 3.2 Randomized Controlled Trial

Of the 40 patients included in the study, 13 women (INT, mean age 55.92 ± 10.56 years; mean remission duration 6.36 ± 9.64 years) completed the program with at least 80% attendance at dance classes, and 10 women (CTRL, mean age 52.70 ± 9.04 years; mean remission duration 7.01 ± 9.50 years) completed the study as controls.

#### 3.2.1 Body Composition, Metabolic Health, and Physical Fitness

After the 12‐week combined dance and dietary intervention, participants in the INT group showed a significant decrease in body weight, BMI, body fat percentage, muscle mass, visceral fat, waist and hip circumference, and WHR compared with CTRL, with large effect sizes (Table 1). Moreover, a significant reduction in GGT and HOMA‐IR was observed in the INT group (GGT pre: 0.50 ± 0.26 ukat/L, post: 0.41 ± 0.18 ukat/L, *p* = 0.003, r(rb) = −1; HOMA‐IR pre: 2.06 ± 1.37, post: 1.70 ± 1.24, *p* = 0.040, r(rb) = −0.697), while no significant changes occurred in the CTRL group (GGT pre: 0.53 ± 0.14 ukat/L, post: 0.55 ± 0.10 ukat/L, *p*‐value = 0.799, r(rb) = 0.091; HOMA‐IR pre: 2.88 ± 2.37, post: 2.60 ± 2.09, *p*‐value = 1.00, r(rb) = 0). At baseline, no significant differences were detected between the INT and CTRL groups for both GGT (*p* = 0.438, r(rb) = −0.192) and HOMA‐IR (*p* = 0.494, r(rb) = −0.169). Lastly, a significant increase in cardiorespiratory fitness (VO_2_max, VEmax, Rfmax, and Loadmax) was observed in the INT group compared with the CTRL group, with large effect sizes. Significant improvements in motor performance (Arm Curl Test and Chair Stand Test) were recorded in both groups. At baseline, no significant differences were detected between the INT and CTRL groups across all measurements (Table 1).

**TABLE 1:** Body composition characteristics and physical fitness characteristics at baseline and after intervention in both groups.

	Difference in INT	*p*‐value within INT	Effect size (r(rb)) within INT	Difference in CTRL	*p*‐value within CTRL	Effect size (r(rb)) within CTRL	*p*‐value between groups (pre‐intervention)	Effect size (r(rb)) between groups (pre‐intervention)
Weight (kg)	−4.04 ± 2.46	**0.002**	−0.978	0.03 ± 1.98	0.799	−0.091	0.710	−0.092
BMI (kg/m^2^)	−1.45 ± 0.87	**0.002**	−0.978	0.02 ± 0.76	0.906	−0.044	0.264	−0.277
Fat (kg)	−3.29 ± 2.20	**0.002**	−0.978	−0.16 ± 1.87	0.333	−0.345	0.620	−0.123
Fat (%)	−1.85 ± 1.58	**0.003**	−0.934	−0.13 ± 1.45	0.440	−0.289	0.352	−0.231
Muscle mass (kg)	−0.49 ± 0.72	**0.037**	−0.679	0.01 ± 0.73	0.635	0.178	0.901	0.031
Visceral fat	−1.39 ± 1.50	**0.011**	−1	0.01 ± 0.57	0.564	0.333	0.676	−0.1
Waist circumference (cm)	−2.42 ± 2.40	**0.007**	−0.964	2.65 ± 7.42	0.332	0.345	0.171	−0.338
Hip circumference (cm)	−4.17 ± 4.18	**0.004**	−0.936	−0.99 ± 5.24	0.474	−0.255	0.367	−0.223
WHR index	−0.03 ± 0.03	**0.002**	−0.956	−0.01 ± 0.18	0.324	−0.389	0.664	0.108
VO2_max_ (ml/min/kg)	3.27 ± 3.24	**0.005**	0.879	0.48 ± 2.49	0.646	0.164	0.352	0.231
VE_max_ (l/min)	12.60 ± 11.59	**0.006**	0.868	0.01 ± 8.17	0.959	0.018	0.321	−0.246
RF_max_ (l/min)	3.60 ± 3.87	**0.006**	0.868	−1.65 ± 5.03	0.445	−0,273	0.264	−0.277
Load_max_ (watt)	18.57 ± 15.92	**0.003**	0.923	4.54 ± 18.47	0.508	0.236	0.686	−0.1
Handgrip Strength Test LH (*N*)	0.94 ± 3.16	0.382	0.275	0.11 ± 3.30	0.760	0.109	0.620	0.123
Handgrip Strength Test RH (*N*)	2.52 ± 3.06	**0.013**	0.78	−0.21 ± 4.25	0.878	−0.054	0.277	0.269
30‐Second Chair Stand Test (*n*)	2.85 ± 4.41	**0.026**	0.758	4.07 ± 5.07	**0.050**	0.733	0.756	0.077
30‐Second Arm Curl Test LH (*n*)	5.91 ± 4.01	**0.001**	1	3.42 ± 4.14	**0.036**	0.745	0.455	−0.185
30‐Second Arm Curl Test RH (*n*)	5.092 ± 3.86	**0.001**	1	3.41 ± 3.39	**0.041**	0.727	0.418	−0.2

*Note:* Values are presented as mean ± SD. Differences were considered significant (in bold) at *p* < 0.05. *p*‐values are reported as unadjusted *p*‐values and should be interpreted as exploratory. Effect sizes were interpreted as small (r(rb) ≈ 0.1), medium (r(rb) ≈ 0.3), and large (r(rb) ≥ 0.5).

Abbreviations: BMI, body mass index; CTRL, control group; INT, intervention group; LH, left hand; Loadmax, maximal workload achieved on the ergometer; RFmax, maximal respiratory frequency; RH, right hand; VEmax, maximal minute ventilation; VO2max, maximal oxygen uptake; WHR index, waist‐to‐hip ratio.

#### 3.2.2 Physical Exercise and Nutrition Reports

Within INT, a significant reduction in total caloric intake, carbohydrate intake, and fat consumption was observed between baseline and during study. In the INT group, measured carbohydrate and protein intake during the intervention was significantly lower than the prescribed values. Furthermore, no significant differences in total caloric or macronutrient intake were detected between the INT and CTRL at baseline. Daily physical activity, excluding the dance classes, did not differ significantly between the INT and CTRL groups at baseline, nor did it change within the INT group during the study (Table 2).

**TABLE 2:** The data analysis of nutrition and physical activity reports.

	INT‐pre	INT‐post	*p*‐value within INT	INT—Prescribed	*p*‐value between prescribed and INT‐post	CTRL	*p*‐value between CTRL and INT‐pre
Energy (kcal/day)	1534.80 ± 391.96	1344.04 ± 277.39	**0.013**	1423.08 ± 107.27	1.186	2425.32 ± 1597.07	0.166
Protein (g/day)	70.20 ± 21.69	73.89 ± 23.92	0.972	89.42 ± 6.45	0.016	64.18 ± 15.05	0.843
Carbohydrate (g/day)	172.90 ± 55.72	144.66 ± 33.22	**0.005**	178.85 ± 12.89	0.006	164.19 ± 35.42	0.905
Lipid (g/day)	58.13 ± 17.57	46.83 ± 15.77	**0.002**	39.74 ± 2.87	0.245	54.01 ± 17.46	0.968
Fiber (g/day)	17.87 ± 6.09	19.84 ± 6.078	0.249	19.82 ± 1.30	0.659	13.79 ± 5.38	0.166

	INT‐pre	INT‐post	*p*‐value within INT	CTRL	*p*‐value between CTRL and INT‐pre
Sedentary activity (h/day)	17.79 ± 1.59	17.21 ± 1.75	0.180	17.38 ± 2.83	0.586
Light physical activity (h/day)	5.63 ± 1.40	6.13 v 1.68	0.180	5.63 ± 2.56	0.783
Vigorous physical activity (h/day)	0.58 ± 0.79	0.67 ± 0.78	0.317	1.00 ± 1.77	0.930

*Note:* Values are presented as mean ± SD. Differences were considered significant (in bold) at *p* < 0.05. *p*‐values are reported as unadjusted *p*‐values and should be interpreted as exploratory. INT‐pre and INT‐post represent actual (self‐reported) intake; INT‐prescribed represents the prescribed dietary intake. CTRL represents actual (self‐reported) intake at baseline.

Abbreviations: CTRL, control group; INT, intervention group.

#### 3.2.3 Gut Microbiota Analysis

**FIGURE 4:**
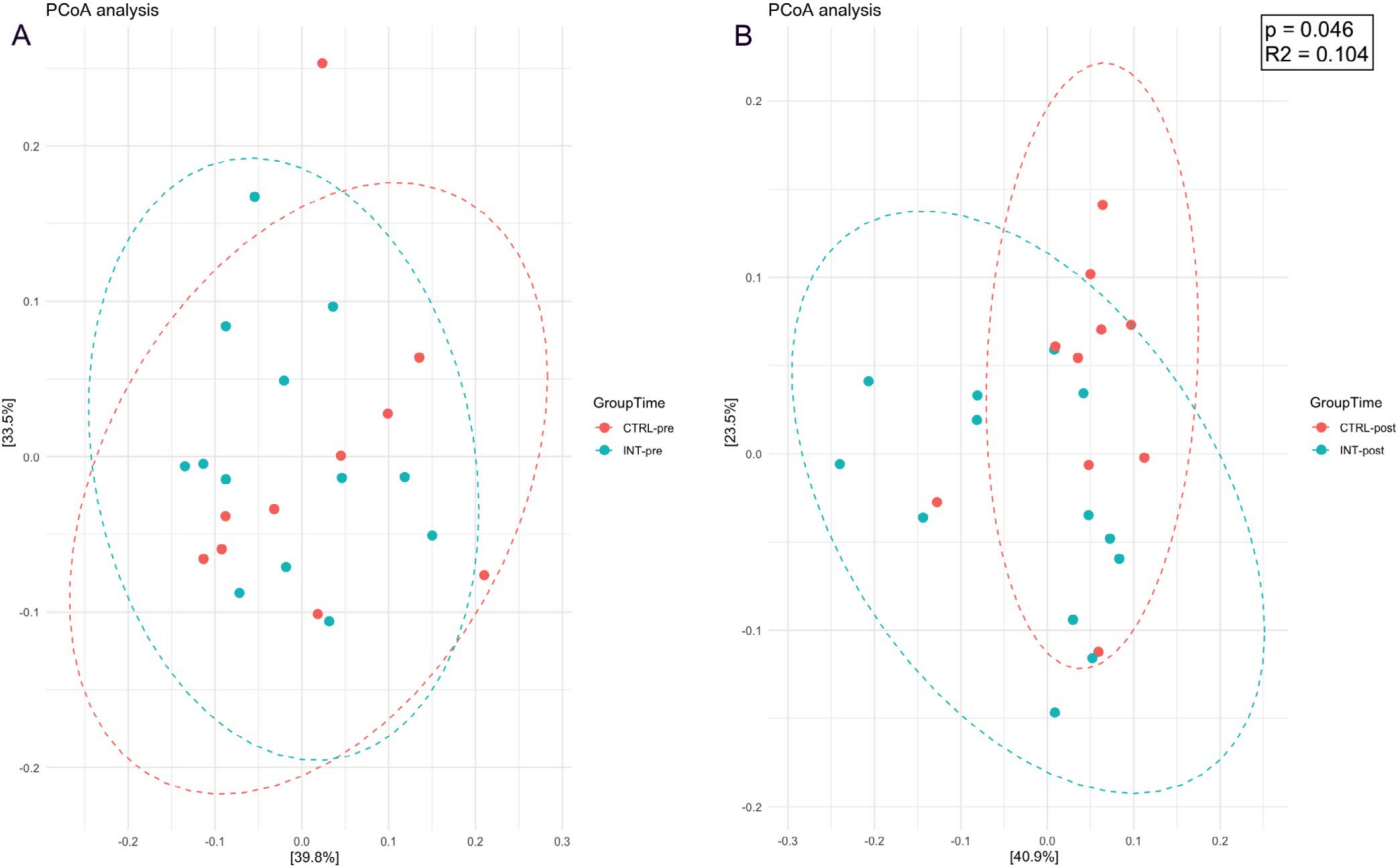
PCoA of gut microbiota beta diversity in the randomized controlled trial based on Bray–Curtis dissimilarity, showing (A) baseline (INT‐pre vs. Ctrl‐pre) and (B) post‐intervention (INT‐post vs. Ctrl‐post) comparisons at the phylum level. Each point represents an individual participant, and dashed ellipses indicate 95% confidence intervals for each group. The percentage of variance explained by the first two principal coordinates is shown on the axes. INT intervention group, CTRL control group.

In the randomized controlled trial, no significant changes in gut microbiota alpha diversity were observed following the intervention in INT group, including Shannon index (*p* = 0.929, r(rb) = 0.030) and Chao1 index (*p* = 0.279, r(rb) = −0.341). However, Chao1 index significantly decreased in CTRL (*p* = 0.037, r(rb) = −0.745). At baseline, no significant differences were observed between INT and CTRL in alpha diversity. In parallel, gut microbiota beta diversity at the phylum level differed significantly across groups and time points (PERMANOVA, *p* = 0.032; *R* ^2^ = 0.083). A significant difference was detected between the post‐intervention INT and CTRL groups in pairwise comparisons (*p* = 0.046, *R* ^2^ = 0.11; Figure 4).

Significant differences after the intervention were detected across 2 phyla, 8 orders, 6 families, 19 genera, and 29 species. The complete results are provided in the Table S2. Among these shifts, a significant decrease in the relative abundance of *Firmicutes* (INT‐pre 74.77 ± 7.59, INT‐post 69.84 ± 7.48; *p* = 0.039, r(rb) = −0.648) was observed. At lower taxonomic levels, the relative abundance of butyrate‐associated taxa, including *Ruminococcus bromii* (INT‐pre 0.44 ± 0.41, INT‐post 0.17 ± 0.16; *p* = 0.023, r(rb) = −0.714) and *Ruminiclostridium hungatei* (INT‐pre 3.05 ± 2.59, INT‐post 1.42 ± 1.35; *p* = 0.028, r(rb) = −0.692), significantly decreased. In contrast, the relative abundance of health‐associated taxa, including *Bifidobacterium pseudocatenulatum* (INT‐pre 0.19 ± 0.40, INT‐post 1.56 ± 3.76; *p* = 0.013, r(rb) = 0.780), *Bifidobacterium animalis* (INT‐pre 0.01 ± 0.02, INT‐post 0.05 ± 0.08; *p* = 0.028, r(rb) = 0.692), significantly increased. Moreover, a significant reduction in opportunistic pathogens, including *Klebsiella oxytoca* (INT‐pre 0.01 ± 0.01, INT‐post 0.00 ± 0.00; *p* = 0.017, r(rb) = −0.944), was detected. No significant changes were observed at baseline between groups or within the CTRL for any of the aforementioned taxa.

#### 3.2.4 Gut Permeability

Following the 12‐week combined dance and dietary intervention, a non‐significant decreasing trend in zonulin concentrations was observed in the INT group (pre: 530.96 ± 402.13 ng/mL; post: 457.80 ± 318.93 ng/mL; *p* = 0.173, r(rb) = −0.429), whereas a significant increase occurred in the CTRL group over the study period (pre: 530.40 ± 106.03 ng/mL; post: 666.48 ± 67.79 ng/mL; *p* = 0.012, r(rb) = 0.714). At baseline, no significant differences were observed between INT and CTRL in zonulin concentrations. Furthermore, significant negative associations were detected between changes in zonulin concentrations and the relative abundance of beneficial bacteria, including *Anaerobutyricum halli* (*p* = 0.049, *r* = −0.555), *Bifidobacterium breve* (*p* = 0.041, *r* = −0.571), and *Streptococcus thermophilus* (*p* = 0.010, *r* = −0.681), whereas significant positive associations were detected between changes in zonulin concentrations and the relative abundance of pathogenic bacteria, including *Proteobacteria* (*p* = 0.024, *r* = 0.621) and *Neisseria* (*p* = 0.039, *r* = 0.577).

#### 3.2.5 Quality of Life

Cronbach's alpha coefficients for the EORTC QLQ‐C30 and EORTC QLQ‐BR23 ranged between 0.70 and 0.90. After the 12‐week combined dance and dietary intervention, significant improvements were observed in the INT group in role functioning (*p* = 0.033; mean change = 1.50 points, 22.4%), fatigue (*p* = 0.006; mean change = 2.46 points, 27.4%), and body image (*p* = 0.019; mean change = 1.96 points, 16.3%). Statistically significant post‐treatment differences between INT and CTRL were detected in role functioning (*p* = 0.025; mean change = 1.95 points, 30%), emotional functioning (*p* = 0.024; mean change = 3.61 points, 27.5%), social functioning (*p* = 0.010; mean change = 2.20 points, 36.7%), fatigue (*p* = 0.003; mean change = 3.45 points, 38.4%), pain (*p* = 0.003; mean change = 2.84 points, 47.3%), and systemic treatment–related side effects (*p* = 0.011; mean change = 3.84 points, 18.3%). At baseline, no significant differences were observed between the INT and CTRL groups across quality‐of‐life domains, except for systemic treatment–related side effects. Table S3 provides the complete data for all quality‐of‐life domains.

## 4 Discussion

This pilot study was designed with two complementary components: a cross‐sectional observational study and a randomized controlled trial. In the observational component, we compared gut microbiota composition among breast cancer patients with obesity, non‐cancer controls with obesity, and healthy controls to identify disease‐related microbial differences. In the randomized controlled trial, we employed breast cancer patients with obesity to explore the effect of a 12‐week combined dance and dietary intervention on the gut microbiota composition, intestinal permeability, body composition, metabolic health, physical fitness, and quality of life. The main findings of our study were the following: (1) breast cancer patients with obesity showed significant negative shifts in gut microbiota compared to both controls, (2) the combined dance and dietary intervention led to favorable changes in body composition, insulin sensitivity, and physical fitness, (3) quality of life improved following the combined dance and dietary intervention, and (4) favorable changes were observed in gut microbiota composition and intestinal permeability following the combined dance and dietary intervention.

Our observational analysis demonstrated more pronounced negative shifts in gut microbiota composition in breast cancer patients with obesity compared with both healthy controls and non‐cancer controls with obesity. Reduced gut microbial α‐diversity has been previously reported in breast cancer patients compared with healthy controls [39, 40], as well as in individuals with obesity compared with normal‐weight controls [41]. Similar patterns were observed in our study, with significantly higher α‐diversity in healthy controls compared with both breast cancer patients with obesity and non‐cancer controls with obesity. However, in this study, no significant difference in α‐diversity was detected between breast cancer patients with obesity and non‐cancer controls with obesity, corroborating previous findings [9]. Nonetheless, significant β‐diversity differences were identified between breast cancer patients and both control groups, while no differences were detected between healthy controls and non‐cancer controls with obesity across all taxonomic levels. These findings suggest that cancer‐related factors, including treatment exposures, may contribute to compositional restructuring of the gut microbiome beyond the diversity reduction associated with obesity alone. This explanation is further supported by studies reporting no significant differences in α‐ or β‐diversity between breast cancer patients with and without obesity [42].

Furthermore, in breast cancer patients, it was found a significantly decrees in relative abundance of *Bacteroidetes* and a significantly increased abundance of *Firmicutes* compared with both control groups, while no significant differences were detected between healthy controls and non‐cancer controls with obesity. This observation is consistent with previous studies reporting higher *Firmicutes* and lower *Bacteroidetes* abundance in breast cancer patients [43, 44]. Although *Firmicutes* increased overall, we observed a reduction in several beneficial bacteria within this phylum. *Faecalibacterium prausnitzii* was significantly decreased in breast cancer patients with obesity compared to non‐cancer controls with obesity. Interestingly, evidence suggests *Faecalibacterium prausnitzii* may suppress the proliferation and invasion of breast cancer cells and promote apoptosis [45].

The combined dance and dietary intervention in our study had favorable effects on body composition, insulin sensitivity, and meaningful improvements in physical fitness in breast cancer patients with obesity. A substantial body of literature supports obesity as a well‐documented risk factor for breast cancer, as demonstrated in a cohort study of 1891 breast cancer female survivors from which 56.1% were reported with obesity and 68.3% with central obesity [46]. After 12 weeks of intervention, we observed significant reductions in BMI, body weight, body fat percentage, and waist and hip circumferences. These findings are consistent with previous post‐exercise effects in breast cancer patients, particularly those involving aerobic, resistance, or combined training modalities [47, 48, 49]. Although promising, evidence from dance‐based interventions in these patients remains limited and has shown mixed effects on body composition outcomes [50, 51, 52]. A possible explanation for these inconsistencies is the lack of detailed dietary intake monitoring. In our study, the beneficial effects of physical exercise on body composition may have been further supported by reductions in total energy intake and lower carbohydrate and fat consumption, consistent with findings from combined exercise–diet interventions in breast cancer patients [53, 54]. Moreover, within the context of metabolic health, the observed reduction in HOMA‐IR following the intervention aligns with previously reported improvements in insulin sensitivity associated with exercise training in these patients [55].

Importantly, cardiorespiratory fitness and muscular strength increased significantly following the combined dance and dietary intervention. Similar improvements in VO_2_max have been reported in previous intervention studies, including dance‐based programs [52, 56], home‐based training [57], and aerobic or resistance exercise interventions in breast cancer patients [58, 59]. In our study, VO_2_max increased by 3.3 mL/kg/min, which is comparable to improvements reported in home‐based interventions (+1.5 mL/kg/min) [57]. However, this increase was smaller than that observed in supervised aerobic or resistance training programs (+9.3 to +11.8 mL/kg/min) [58, 59]. Moreover, our findings on muscular strength improvements correspond with previous reports showing improvements in [52, 56, 60], as reflected by higher values in handgrip strength, arm curl performance, and the 30‐s chair stand test after 12‐week intervention. These improvements are clinically relevant, as physical function has been shown to predict survival and mortality in breast cancer patients [61]. Importantly, physically active women with breast cancer may achieve levels of cardiorespiratory fitness and muscular strength comparable to those of physically active healthy women [62].

Notably, breast cancer patients participating in the intervention demonstrated significant differences in several quality‐of‐life domains compared with controls. Significant effects were observed particularly in role, emotional, and social functioning, domains previously shown to respond to combined training [63, 64, 65] and dance‐based interventions [52] in breast cancer survivors. In the symptom scales, the post‐intervention group showed lower fatigue and pain compared with the control group, corroborating earlier findings [52, 63, 65, 66]. This is clinically relevant, as fatigue and pain are among the symptoms with the greatest negative impact on quality of life following cancer treatment [67]. However, the evidence regarding pain remains equivocal, as data from a meta‐analysis [64] reported no significant overall effect of exercise in breast cancer patients. After 12‐week intervention we observed a significant improvement in body image compared with controls, consistent with prior research in breast cancer populations [65, 66]. Participation in group‐based exercise may have contributed to enhanced body image perception and overall well‐being by fostering social interaction, shared experiences, and peer support among participants [66]. Collectively, these findings further support the growing body of evidence suggesting that combined dance and dietary intervention can meaningfully contribute to quality of life in women recovering from breast cancer [68, 69]. Despite the relatively small sample size, the intervention demonstrated statistically significant improvements across multiple quality‐of‐life domains, highlighting its promising clinical potential as a supportive strategy in breast cancer care.

The 12‐week combined dance and dietary intervention induced favorable changes in gut microbiota composition at the phylum level measured by beta diversity. This significant separation between groups at post‐intervention suggests combined dance and dietary intervention may influence microbial composition without necessarily affecting total diversity. This observation aligns with emerging evidence that exercise more commonly induces compositional remodeling rather than broad increases in diversity [70]. Exercising two to three times per week appears sufficient to influence beta diversity, whereas four to five weekly sessions are typically required to elicit changes in alpha diversity [71]. This observation is also supported by the evidence indicating physical activity and higher cardiorespiratory fitness are associated with greater microbial diversity in breast cancer survivors [11, 72]. In contrast, a significant decrease in alpha diversity was observed in the CTRL group, which may reflect natural temporal or seasonal variation in gut microbiota composition, as previously reported in the literature [73].

Several notable microbial shifts were found following the intervention, including a significant decrease in the relative abundance of *Firmicutes* in the intervention group. Although *Firmicutes* comprise a large proportion of commensal gut bacteria, alterations in the *Firmicutes‐to‐Bacteroidetes* balance have frequently been associated with metabolic regulation and obesity‐related phenotypes [74]. Therefore, the observed reduction of *Firmicutes* may reflect a shift toward a microbial profile linked to improved metabolic health, although functional implications require further investigation. Unexpectedly, we observed a decrease in several butyrate‐producing taxa, including *Ruminococcus bromii* and *Ruminiclostridium hungatei*. These bacteria are known contributors to resistant starch degradation and short‐chain fatty acid (SCFA) production [75]. While this contrasts with the expected beneficial effects of exercise, it may reflect a specific ecological response to the combined dance and dietary intervention, or it could be a chance finding due to multiple comparisons. Future studies with metabolomics are needed to determine if total butyrate production was affected.

Importantly, the intervention promoted significant increases in several widely recognized beneficial bacterial strains, including *Bifidobacterium pseudocatenulatum* and *Bifidobacterium animalis* . Members of the *Bifidobacterium* are known to modulate host immune function, interact with the intestinal mucosa, and produce SCFAs that contribute to cross‐feeding interactions and gut barrier function within the gut ecosystem [76]. Impaired barrier function and inflammation have been reported in breast cancer patients [77]. While zonulin, referred to as gut barrier function [78], showed a non‐significant decrease in the intervention group, it increased significantly in controls. This may partly reflect natural temporal or seasonal variation in gut microbiota and related host markers, potentially contributing to the unfavorable shifts observed in the control group [73]. However, serum zonulin concentration may not fully reflect intestinal permeability [79]. Furthermore, positive associations were detected between zonulin changes and potentially pathogenic bacteria, including *Proteobacteria* and *Neisseria*. *Proteobacteria* expansion is commonly considered a hallmark of dysbiosis and has been linked to inflammation and colonic epithelial dysfunction [80]. Additionally, a significant reduction of the opportunistic pathogen *Klebsiella oxytoca* was observed following the intervention. This species has been linked to cancer cachexia and inflammatory conditions [81], and its decrease may represent a clinically meaningful improvement in microbial balance.

A key strength of this study is the comprehensive evaluation of the effects of a combined dance and dietary intervention in breast cancer patients with obesity, including gut microbiota, intestinal permeability, body composition, metabolic blood parameters, cardiorespiratory fitness, muscular strength, and quality of life. Moreover, to our knowledge, this is the first human study examining the combined effects of physical activity and dietary intervention on the gut microbiome in breast cancer patients. In addition, our study included a comparison between breast cancer patients with obesity and non‐cancer controls with obesity, whereas most previous studies have compared cancer patients primarily with healthy individuals.

This study has several limitations. First, the use of a combined dance and dietary intervention prevents distinguishing the independent effects of each component. Second, a higher‐than‐expected dropout rate led to a reduced final sample size, which may have limited the statistical power to detect smaller effects. Third, microbiota profiling was performed using 16S rRNA gene sequencing targeting the V3–V4 region, which limits taxonomic resolution and does not provide direct functional insights compared with whole‐genome shotgun metagenomic approaches.

## 5 Conclusion

In conclusion, breast cancer patients with obesity exhibited distinct gut microbiota alterations compared to both non‐cancer controls with obesity and healthy controls. In addition, the 12‐week combined dance and dietary intervention led to significant improvements in body composition, insulin sensitivity, cardiorespiratory fitness, muscle strength, and quality of life. The intervention was also associated with select changes in gut microbiota, including an increase in *Bifidobacterium* species. However, a decrease in butyrate‐producing taxa was also observed. Larger, adequately powered trials with diet‐only control groups and metagenomic sequencing are needed to clarify the specific role of physical activity in modulating the gut microbiome in breast cancer survivorship.

## Author Contributions

**Adela Penesová:** conceptualization, project administration, investigation. **Jenni Hekkala:** investigation. **Simona Ugrayová:** investigation. **Satu Pekkala:** investigation, writing – review and editing. **Viktor Bielik:** writing – review and editing, resources, conceptualization, funding acquisition, methodology. **Ivan Hric:** formal analysis, data curation, visualization. **Miroslava Šimiaková:** investigation. **Libuša Nechalová:** writing – original draft, formal analysis, data curation. **Peter Olej:** investigation. **Simona Kelčíková:** investigation, data curation. **Marián Grendár:** data curation.

## Funding

This work was supported by the Ministry of Education, Research, Development and Youth of the Slovak Republic (APVV‐22–0047), (APVV‐23‐0028), (APVV booster 09I03‐03‐V06‐00062), and (VEGA 1/0313/25).

## Conflicts of Interest

The authors declare no conflicts of interest.

## Supporting information


**Table S1:** Data analysis of all taxonomic differences among the three study groups in the cross‐sectional observational study.
**Table S2:** Analysis of taxonomic differences within intervention group.
**Table S3:** Analysis of quality‐of‐life domains measured using the EORTC QLQ‐C30 and EORTC QLQ‐BR23 questionnaires.

## Data Availability

The data that support the findings of this study are openly available in NCBI SRA database at https://www.ncbi.nlm.nih.gov/sra, reference number PRJNA1424373.
